# Interaction of HuDA and PABP at 5'UTR of mouse insulin2 regulates insulin biosynthesis

**DOI:** 10.1371/journal.pone.0194482

**Published:** 2018-03-28

**Authors:** Poonam R. Pandey, Rucha D. Sarwade, Abdul Khalique, Vasudevan Seshadri

**Affiliations:** 1 National Centre for Cell Science, Ganeshkhind, Pune, India; 2 Department of Biotechnology, Savitribai Phule Pune University, Ganeshkhind, Pune, India; University of Louisville, UNITED STATES

## Abstract

Understanding the regulation of insulin biosynthesis is important as it plays a central role in glucose metabolism. The mouse insulin gene2 (Ins2) has two splice variants; long (Ins2L) and short (Ins2S), that differ only in their 5’UTR sequence and Ins2S is the major transcript which translate more efficiently as compared to Ins2L. Here, we show that cellular factors bind preferentially to the Ins2L 5’UTR, and that PABP and HuD can bind to Ins2 splice variants and regulate its translation. *In vitro* binding assay with insulin 5’UTR and different HuD isoforms indicate that the ‘N’ terminal region of HuD is important for RNA binding and insulin translation repression. Using reporter assay we showed that specifically full-length HuD A isoform represses translation of reporter containing insulin 5’UTR. We further show that PABP and HuD interact with each other in RNA-dependent manner and this interaction is affected by glucose and PDI (5’UTR associated translation activator). These results suggest that PABP interacts with HuD in basal glucose conditions making translation inhibitory complex, however upon glucose stimulation this association is affected and PABP is acted upon by PDI resulting in stimulation of insulin translation. Together, our findings snapshot the mechanism of post-transcriptional regulation of insulin biosynthesis.

## Introduction

Insulin is a small peptide hormone that plays a crucial role in energy metabolism in vertebrates and is responsible for regulating glucose homeostasis in the body. Glucose stimulated insulin translation is a well studied phenomenon. Glucose regulates several steps of insulin biosynthesis starting from transcription, splicing, export, stability, and translation resulting in 20 fold increase in insulin production [1–3] but the reason for the apparent quiescence in insulin translation at low/basal glucose level is still unclear. The insulin2 gene of rodents contains two introns while the insulin1 gene contains only one intron and is believed to have evolved by retrotransposon-mediated gene duplication with loss of intron 2 [4,5]. We have previously reported the expression of a novel splice variant for the mouse insulin2 (Ins2) [6]. Alternative splicing in the untranslated regions (UTR) can affect mRNA translation, stability or localization which may be regulated by specific RNA binding proteins (RBP’s) [7]. The alternative splicing of Ins2 mRNA results in significant changes to the 5’UTR sequence and structure and these splice variants have different translatability with the long variant being less translatable [6]. Results also suggest that the 12 additional bases present in long 5’UTR mediate differential binding of *trans-acting* factors/miRNA [6,8].

We have previously identified Protein-Disulfide Isomerase (PDI) as a key factor responsible for glucose stimulated insulin biosynthesis by bringing disulfide bond modification in poly-A-binding protein (PABP) [9]. PABP exhibits a stimulatory effect on translation by forming a closed loop where it acts as a *cis*-*acting* effector molecule [10,11]. The role of PABP is also well characterized in polyadenylation, export and turnover of the transcripts [12].

It has also been shown that PABP can bind to ‘A’ rich region in the 5’UTR of its own mRNA and represses the translation [13]. Apart from binding to its own mRNA, PABP also binds to ‘A’ rich sequence present within the 5′ UTR of scaffold attachment Factor B (Safb) [14], a protein associated with cell and tumor growth, metabolism and obesity [15]. More recently, transcriptome-wide CLIP-Seq analysis has shown that PABP can bind to ‘A’ rich sequence in 5’UTR of several transcripts and in many instances results in translation repression [16]. However, the role of PABP in insulin translation by binding to its 5’UTR is still obscure.

Human antigen D (HuD) binds to mouse Ins2 5’UTR and represses insulin translation [17,18]. Previous studies identified several HuD isoforms in neuronal cells, generated due to alternative splicing [19–21]. Further, it was suggested that the unique sequences in ‘N’ terminal and the linker region (site of PTM-phosphorylation) of HuD play a significant role in RNA binding activity [22,23]. Individual isoforms of HuD perform specific functions such as splicing or stabilizing transcripts by binding to ARE region [24,25], but the exact mechanism of isoform specific translation regulation of insulin in pancreatic cell is not known. Here, in this report, we show that PABP interacts efficiently with long variant (Ins2L) of insulin2 mRNA along with HuD. We found that HuD A, a specific isoform of HuD represses the translation of insulin long variant. Further, PABP and HuD interacts in RNA dependent manner. Interestingly, this interaction is diminished in glucose stimulated condition or in the presence of activator molecule PDI. Together, our findings highlight the possible mechanism of insulin translation repression involving PABP and HuD as a part of the complex which plays a significant role in controlling the translation of distinct insulin mRNA splice variants.

## Materials and methods

### Cell culture

βTC6 and MIN6 cells were maintained in Dulbecco’s modified Eagle’s medium (DMEM-Gibco) in low glucose (1g/l) supplemented with 10% FBS (Gibco), 100 μg/ml penicillin and 100 μg/ml streptomycin, under 5% CO_2_ at 37 °C. For glucose stimulation, cells were treated with serum free DMEM medium containing 20 mM glucose.

### Cloning of HuD isoforms and purification of recombinant proteins

Mouse HuD B isoform (NM_001038698.1) and HuD D isoform (NM_001163397.1) ORFs were amplified from βTC6 cDNA by PCR using gene specific primers (S1 Table) containing restriction sites for enzymes *BamHI* and *XhoI* (New England Biolabs) respectively.

HuDA isoform (NM_010488.4) was cloned by replacing the N-terminal of HuD B clone with the N-terminal end of HuDD clone. All three isoforms were also subcloned into pGEX6P3 to make GST-fusion and for mammalian expression vectors into pCDNA3.C3 (Thermo Fisher Scientific) vector having Myc and HA tag at the 5’ end. All clones were confirmed by sequencing.

The clones expressing the His-PABP and His-HuD/GST-HuD isoforms were inoculated into LB-kanamycin and LB-ampicillin media respectively. When the OD of both the cultures reached around 0.5–0.6, IPTG (Sigma) was added at the final concentration of 1 mM for PABP and 0.1 mM for HuD. Further, both cultures were incubated at 37°C and 25°C for 3 h and 5 h respectively. The recombinant proteins were purified by following standard protocols of affinity tag purification (GE healthcare).

### Competitive RNA-EMSA and UV crosslinking assay

RNA-EMSA was performed as described [6]. Briefly, insulinoma cell extract (βTC6/MIN6) or recombinant purified proteins (PABP/HuD A/HuD B/HuD D) were incubated with radiolabeled RNA probe along with specific competitors. The RNA-protein complex was resolved on a 6% native PAGE in 0.5 X TBE at 4°C. The gel was dried and the complex formation was assessed by autoradiography.

For ultraviolet (UV) crosslinking, RNA-EMSA reactions were exposed to UV light followed by RNase treatment as described [9] and analyzed on 10% SDS PAGE followed by autoradiography.

### RNA immunoprecipitation

RIP assay was performed as described [26]. Briefly, βTC6 cells were exposed in UV, lysed in NT2 (50 mM Tris, 150 mM NaCl, 2 mM MgCl_2_, 0.1% NP40) buffer as described earlier and the lysates (about 500 μg/ 800 μl per RIP reaction) were precleared with protein G beads (Upstate) for 1 h and subjected to immunoprecipitation with either control or specific antibody (4 μg of rabbit anti-PABP antibody (Abcam-21060) and anti-HuD antibody (Santa cruz Biotechnology-H300) at 4°C for 10 h. The beads were blocked in a buffer with denatured t-RNA (Sigma) for 90 min on ice with glycogen (Ambion) and salmon sperm DNA (20 μg) in 100 μl before adding the immunoprecipitate. After incubation, beads were extensively washed in NT2 buffer and the RNA associated with the immunoprecipitate was extracted and analyzed by RT-PCR (Primers listed in S1 Table).

### Biotin pull-down assay

Biotinylated long RNA (5 μg) was incubated with 100 μl of streptavidin agarose beads (Invitrogen) for 30 min then washed thrice with 1X GSB (5 mM Tris, pH 7.5, 15 mM KCl, 5 mM MgCl_2_, 0.25 mM DTT, 20 U of RNasin and 10% glycerol). The 10-fold molar excess of unlabeled long (L) and short (S) RNA were added to RNA bound beads, followed by the addition of the extract (500 μg/mL of reaction) and incubated at 4°C for 2 h. After washing with 1X GSB bound fractions were eluted in 2X-SDS loading dye. The protein complex was resolved on a 10% SDS-PAGE. The gel was transferred on PVDF membrane (Millipore) and probed for PABP (Abcam-21060) and HuD (Santa cruz Biotechnology-H9).

### *In vivo* and *in vitro* translation assay

MIN6 cells (5 x 10^6^) were seeded into 24 well plate and after 16 h media were replaced with DMEM-LG containing 0.1% FBS for 8 h followed by transfection with pcDNA3 L-Luc/ S-Luc construct (450 ng), pcDNA3 renilla luciferase (50 ng) and pcDNA3-HA HuD isoforms such as A, B and D (1 μg each). After 12 h of post transfection cells were recovered for 16 h and then treated with low and high glucose as per standard protocol. The cells were lysed in passive lysis buffer (Promega) after 48 h of post-transfection. For *in vitro* translation assay, 50 ng of cRNA (non-polyadenylated insulin 5’UTR-luciferase-3’UTR) was translated in rabbit retic lysate (RRL) in presence or absence of recombinant proteins (200 ng) as per manufacturer’s protocol (Promega). Renilla-luciferase (15 ng) was included in all reactions as a translation efficiency control. In decoy experiments, competitors were added up to 250 fold molar excess to the luciferase RNA. The luciferase activity was measured with the Dual-Luciferase Reporter Assay System (Promega Corp.).

### Insulin secretion assay and insulin immunobloting

HuD isoform (2.5 μg) plasmids were transfected in MIN6 cells and after 24 h of recovery, the cells were treated with low and high glucose media for 30 min. The insulin production was estimated from culture supernatant by ELISA (Millipore EZRMI-13K) following the instruction manual and the intracellular insulin levels were also assessed by probing with anti-insulin antibody (Santa Cruz Biotechnology-9160).

### Immunoprecipitation and *in vitro* binding assays

The extracts prepared in RIPA buffer were subjected to IP, a portion of extracts were also subjected to RNase treatment (Ambion) at 37°C for 10 min before IP. Rabbit polyclonal anti-PABP antibody (Abcam) or rabbit polyclonal anti-HuD antibody (Santacruz Biotechnology-H300) was added to the extracts along with separate reaction of rabbit IgG control for overnight immunoprecipitation followed by binding to agarose protein G beads (Upstate) for 2 h at 4°C.

Ni-NTA and GST-pull down were performed as per standard protocol of affinity tag purification. The (co)-purified proteins were resolved on 10% SDS PAGE and analyzed by western blotting or stained with Coomassie to see efficiency of pull-down.

To examine the interaction between purified proteins equal amount of recombinant His-PABP, His-HuD A and His-GFP (2 μg of each) were mixed and subjected to IP. The recombinant His-PDI, or its C-terminal deletion mutant or the long 5’UTR RNA (150 ng) was also added to the protein mixture before IP and further analyzed by western blotting.

### Secondary structure prediction

The Secondary structure of the RNA was predicted using the m-fold algorithm [27].

### Statistical analysis

Quantitative data are represented as the mean ± SEM and compared statistically by Student’s t test, using Sigma plot (10.0). The p-value of < 0.05 was considered statistically significant and indicated in the figures.

## Results

### Factors from βtc6 extract show differential affinity for splice variants and binds specifically in middle and distal region of Ins2 5’UTR

**Fig 1:**
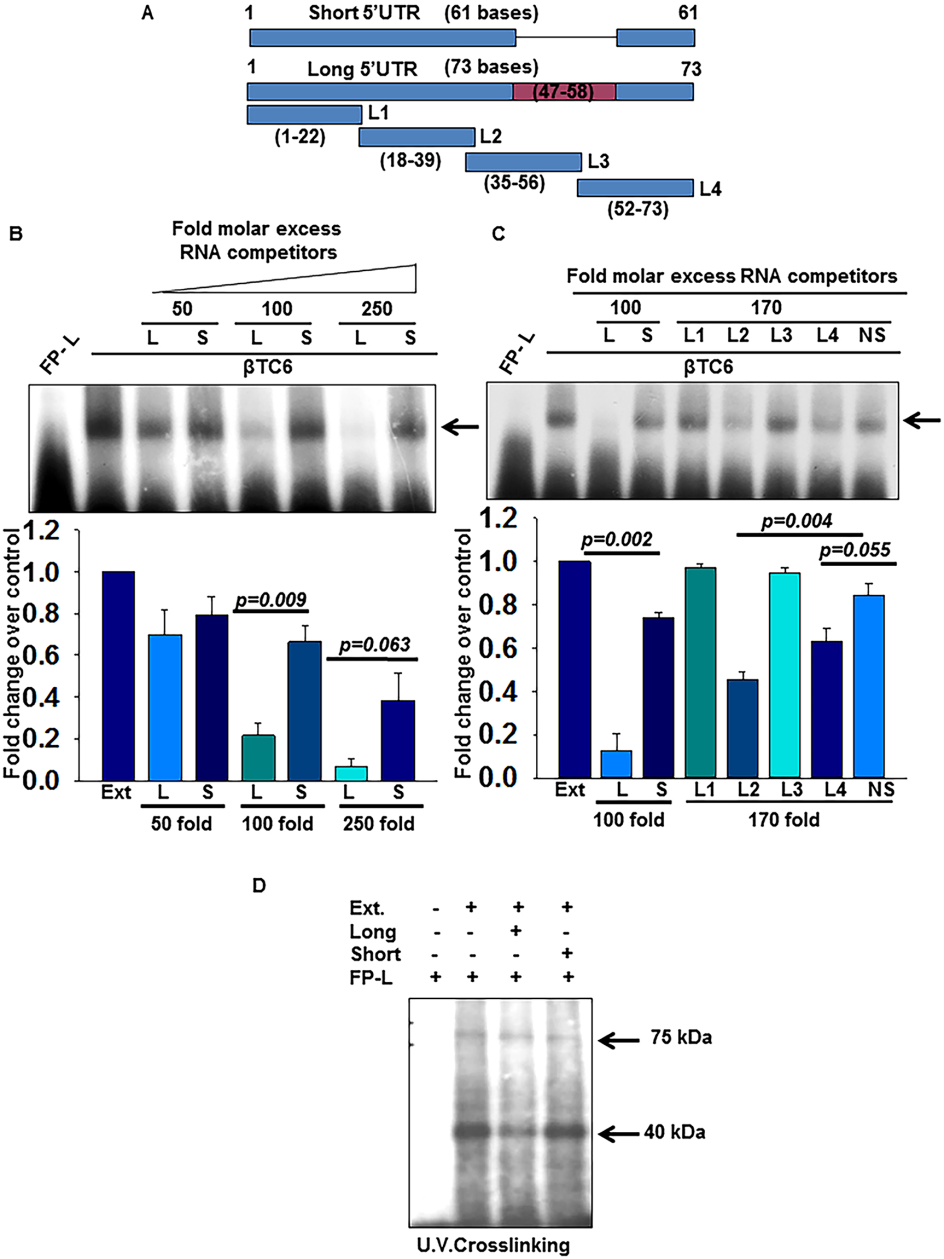
Factors from βTC6 extract bind differentially to Ins2L and Ins2S splice variants. **(A)** Schematic of Ins2 splice variants-Long (L) and Short (S) along with fragments of the long variant (L1-L4). Shaded area depicts the spliced sequences absent in the short 5’UTR. **(B)** Competitive RNA-EMSA using radiolabeled long 5’UTR were performed with βTC6 extracts in presence of the excess of unlabeled long (L) or short (S) RNA and **(C)** in presence of excess of L1, L2, L3, L4 or non-specific RNA (NS) RNA as indicated. The shifted band was quantified and analyzed densitometrically and represented as the bar graph (lower panel). The values are an average of three independent experiments (biological replicates) with error bars representing the mean ± SEM and the significance indicated by exact p values. **(D)** A βTC6 extract was incubated with radiolabeled Ins2L 5’UTR along with excess of unlabeled long and short competitors. The reaction was then exposed to UV radiation and the crosslinked complexes were resolved on SDS-PAGE after RNase digestion.

We have previously shown that long variant of mouse insulin gene 2 (Ins2L) is less efficiently translated as compared with short variant [6]; we postulated that this repression is due to a strong affinity of repressor molecules at 5’UTR. To characterize the RNA binding region in presence of cell extracts, long and short 5’UTR of insulin gene 2 along with RNA fragments of long 5’UTR were synthesized and RNA-EMSA was performed (Fig 1A). The Long and Short 5’UTR RNA sequences are shown in S1A Fig. As expected, factors bind efficiently to radio labeled long variant and addition of molar excess of long unlabeled RNA (L) significantly diminished the intensity of the specific complex. Interestingly, the complex is competed out less efficiently when short 5’UTR (S) is used as a competitor (Fig 1B). Similarly, the radiolabeled short 5’UTR formed a weak complex as compared to long variant and complex was competed out very efficiently with the excess of long or short RNA, indicating the low affinity of *trans-acting* factors to short variant (S1B Fig). Further, in order to determine the specific binding region, we subdivided 5’UTR of Ins2L into four fragments; L1, L2, L3 and L4 as shown in Fig 1A and competitive RNA-EMSA was performed by incubating the lysate with molar excess of unlabeled long 5’UTR probe and its fragments (L1, L2, L3 and L4). We found that RNA corresponding to L2 and L4 region of the insulin long 5’UTR competed efficiently (Fig 1C). Sequence analysis indicates that L2 region (18–39) is about 50% ‘A’ rich with stretch of 5‘A’ residues, while L4 region (52–73) is slightly AU rich (S1C Fig). The complex was not competed out by RNA corresponding to L1 or L3 region as well as by the nonspecific unlabeled RNA (NS) when used as competitors. This led us to conclude that L2 and L4 regions are recognized by the binding factors in the βTC6 lysate, suggesting that more than one factor may associate with insulin 5’UTR at two different regions. In order to test this, we performed UV crosslinking experiments and observed two major crosslinked bands at around 45 kDa and 75 kDa (Fig 1D) indicating that at least two proteins associate with the insulin 5’UTR.

### PABP and HuD associate with the splice variants of Ins2 at 5’UTR

**Fig 2:**
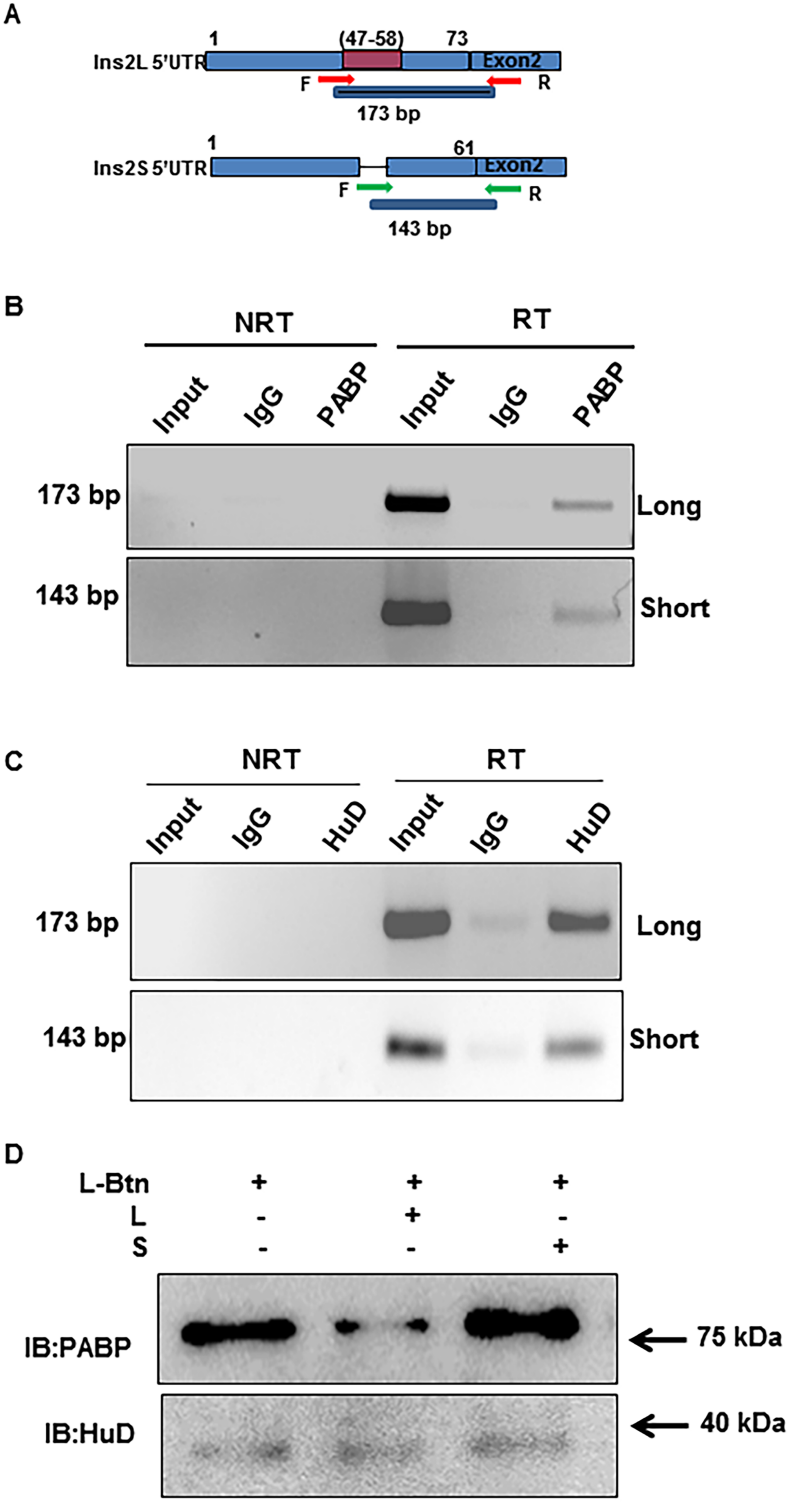
Cellular PABP and HuD associates with mouse insulin transcripts. **(A)** Schematic showing the regions of amplification of Ins2L and Ins2S with the indicated primers. RNA associated with PABP/ HuD specifically immunoprecipitated from UV cross-linked samples were analyzed by RT-PCR using insulin gene specific primers. IgG is used as control. Insulin 5’UTR RNA associated with **(B)** PABP or **(C)** HuD is analyzed by RT-PCR using Ins2L or Ins2S specific primers. Insulin transcript pulldown efficiency with respective proteins (PABP and HuD) is normalized with respect to input used for the experiment. **(D)** Analysis of mouse insulin 5’UTR associated proteins in βTC6 cell lysates using biotinylated Ins2L 5’UTR RNA. The biotin pull-down is carried out in the presence of 10-fold molar excess of non-biotinylated long (L) or short (S) 5’UTR RNA (as competitors) and PABP/HuD associated with insulin RNA was analyzed by western blotting.

PABP and HuD have been shown to bind to insulin 5’UTR leading us to hypothesize that these two could be the two cross linked RNA binding proteins that we have detected in our UV crosslinking experiments. To assess endogenous binding of PABP or HuD to mouse insulin RNA, we performed UV crosslinked RNA Immunoprecipitation (RIP) followed by RT-PCR. A specific amplification of 108 bp corresponding to mouse insulin was observed in input as well as in PABP or HuD immunoprecipitate, but not in IgG control (S2A–S2C Fig). We further verified this association with respect to long and short insulin splice variants using isoform specific primers (Fig 2A) and found that both are specifically enriched in PABP and HuD IP relative to IgG control (Fig 2B and 2C). These results suggest that PABP and HuD associate with mouse Ins2 splice variants. Using biotinylated insulin 5’UTR RNA and unlabelled RNA as competitors we assessed the relative binding of endogenous PABP or HuD to insulin splice variant 5’UTRs. We find that biotinylated insulin 5’UTR RNA was able to pull down both PABP and HuD efficiently. Presence of unlabelled RNA corresponding to Ins2L reduced the amount of PABP associated with the biotinylated RNA when compared to Ins2S or control. These results showed that PABP preferentially associates with long 5’UTR RNA, however, we find that HuD association with biotinylated Ins2L 5’UTR is not significantly affected by the presence of competitors (Fig 2C). Further, direct binding experiments with long and short biotinylated RNA shows that the higher amount of PABP associates with long biotin RNA as compared to short biotin RNA using similar amounts of extract (S2D Fig), indicating that PABP binds differentially to the splice variants of Ins2.

### PABP binds efficiently to the middle region of Ins2L 5’UTR

**Fig 3:**
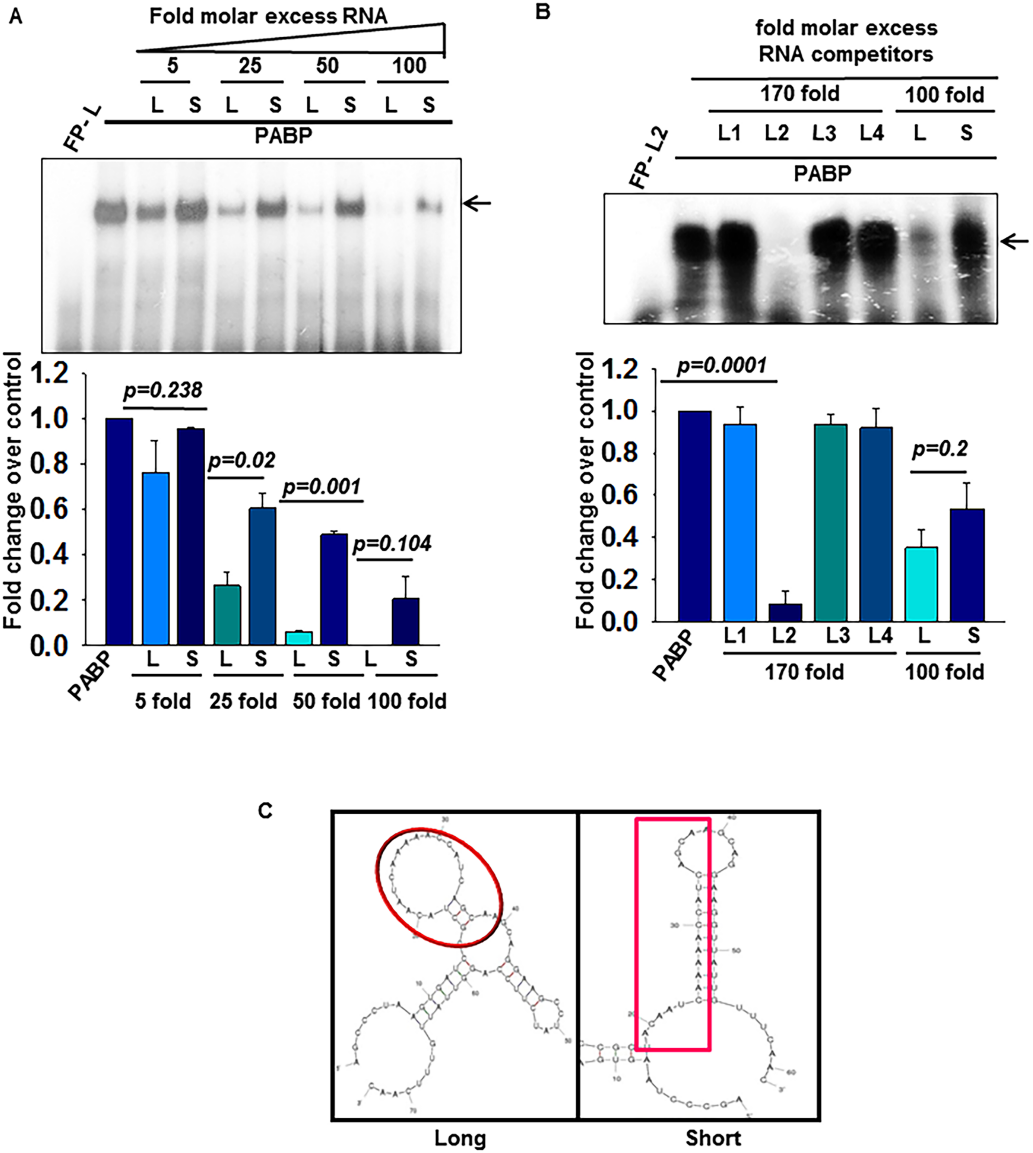
PABP binds differentially to the middle region (L2, position 18–39) of insulin 5’UTR. Competitive RNA-EMSA was performed with recombinant PABP and **(A)** radiolabeled Ins2L-5’UTR RNA as probe in presence of increasing fold excess of unlabeled L or S RNA and **(B**) radiolabeled L2 (position 18–39) RNA as probe in presence of molar excess of unlabeled RNA (L, S, L1, L2, L3, L4). The shifted band was quantified and analyzed densitometrically and represented as the bar graph (lower panel). The values are an average of three independent experiments with error bars representing the mean ± SEM, and the significance indicated by p values. (**C**) m-fold predicted secondary structure of Ins2 splice variants (L/S). The boxed area shows the different folding of L2 region in both the splice variants.

In order to define the region in the 5’UTR RNA to which PABP interacts, we performed competitive RNA-EMSA with recombinant PABP (S3A Fig), radiolabeled Ins2L RNA and RNA fragments of Ins2L as unlabelled competitors. A specific complex was formed with radiolabeled Ins2L RNA and His-PABP which is competed out by molar excess of unlabeled Ins2L RNA as compared to similar fold excess of short unlabeled RNA (Fig 3A). Our data had shown that L2 and L4 region of the insulin 5’UTR associates with specific factors in insulin producing cells (Fig 1C). We find that the specific association of L2 region with PABP, by RNA-EMSA where a specific complex was detected using radiolabeled L2 RNA probe and recombinant PABP (Fig 3B). We also confirmed that only L2 fragment was able to compete out the complex formed between radiolabeled Ins2L RNA and PABP while L1, L3 and L4 RNA fragment was unable to do so (S3B Fig). Taken together, these results demonstrate that L2 region of insulin 5’UTR is the target binding site of PABP.

L2 region is present in both long and short insulin 5’UTR and conserved across species (S3C Fig), however, PABP is predominantly associated with long insulin 5’UTR suggesting that not only the sequence but the context of the sequence may contribute to specific association of PABP to insulin 5’UTR. Secondary structure prediction by m-fold suggests that the L2 region including the stretch of ‘A’ residues lies in single stranded loop region in the Ins2L 5’UTR, however the same region forms double stranded stem in Ins2S 5’UTR (Fig 3C, shaded area). This suggests that the single stranded nature of the L2 region in the long variant may facilitate efficient binding of PABP.

### HuD A/D binds to Ins2 splice variant and associates to both middle and distal region of Ins2L 5’UTR

**Fig 4:**
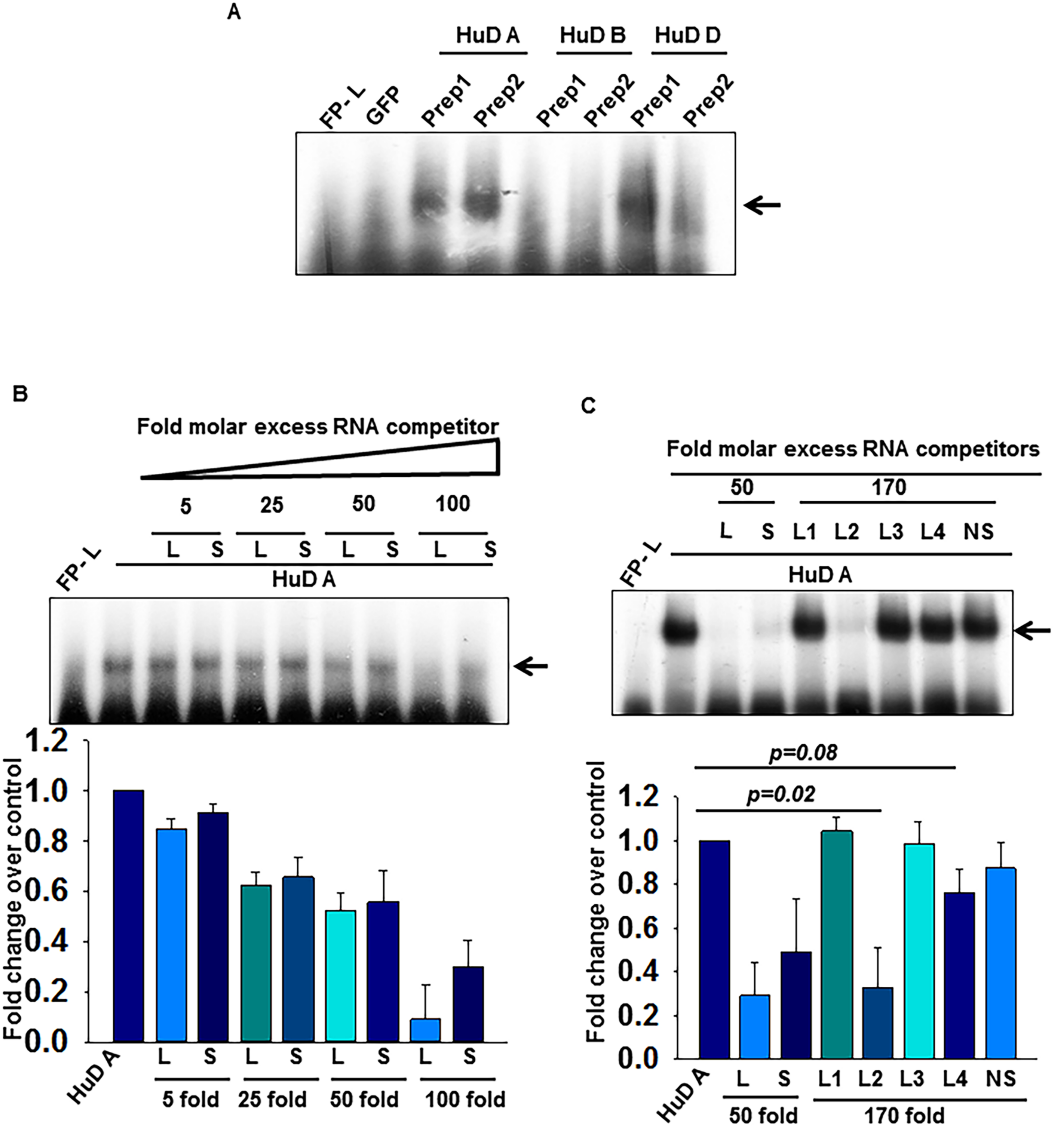
‘N’ terminal of HuD isoform is important for RNA binding. **(A)** RNA-EMSA of two different preparations of HuD isoforms. **(B)** Competitive RNA-EMSA with His-HuD A and radiolabeled Ins2L 5’UTR RNA in presence of increasing molar excess of unlabeled L and S RNA and **(C**) in presence of molar excess of fragments of Ins2L 5’UTR; L1, L2, L3, L4 or non-specific RNA (NS). The shifted band was quantified and analyzed densitometrically and represented as the bar graph (lower panel). The values are an average of three independent experiments with error bars representing the mean ± SEM and the significance indicated by *p* values.

Recently, it was shown that HuD binds to insulin 5’UTR and regulates its translation [17]. We cloned HuD and performed RNA-EMSA to study its binding ability to Ins2 splice variants and we observed that the recombinant HuD was unable to bind to Ins2L 5’UTR RNA. Protein sequence alignment suggests that mouse has different HuD isoforms where HuD-A and HuD-D are identical except the linker region which is missing in HuD-D whereas HuD-A and HuD-B are identical except HuD B has first 7 amino acids different at ‘N’ terminal (S4A Fig). We had cloned the HuD-B isoform initially and did not see any RNA binding activity at insulin 5’UTR hence we cloned, expressed and purified the A and D isoforms as well (S4B–S4D Fig) to assess their binding potential to insulin 5’UTR by RNA-EMSA. A specific complex was formed between Ins2L RNA with His-HuD A and His-HuD D isoform but not with His-HuD B as previously mentioned (Fig 4A). The previous report suggests that RRM 1 and RRM 2 of HuD is crucial for RNA binding activity [28,29], and our results suggest that ‘N’ terminal present at RRM 1 might be playing an important role in RNA binding activity of HuD which is also consistent with the previous finding [19]. We also expressed HuD-B as a GST fusion protein to verify if a different tag would help in restoring the RNA binding activity (S4E Fig) if at all it is misfolded due to His-tag. We find that even GST-HuD B fusion protein has no detectable RNA binding activity with respect to insulin 5’UTR (S4F Fig).

To assess the binding ability of HuD isoforms A/D with respect to splice variants, we performed RNA-EMSA in the presence of unlabeled long and short RNA as competitors. Both HuD-A and D isoforms formed specific complex and was competed out with unlabeled long and short 5’UTR with similar efficiency at lower fold excess competitors, but at higher fold excess there seems to be a differential association suggesting that HuDA and D can bind differentially to the insulin 5’UTR splice isoform at higher concentration of RNA (Fig 4B and S4G Fig). Also, competition with fragments of 5’UTR suggests that these isoforms can associate preferentially with L2 and L4 region of the 5’UTR of insulin (Fig 4C and S4H Fig). These results suggests that L2 region is the common binding site for both PABP and HuD while L4 region can be bound only by HuD.

### HuD A isoform translationally represses Ins2L

**Fig 5:**
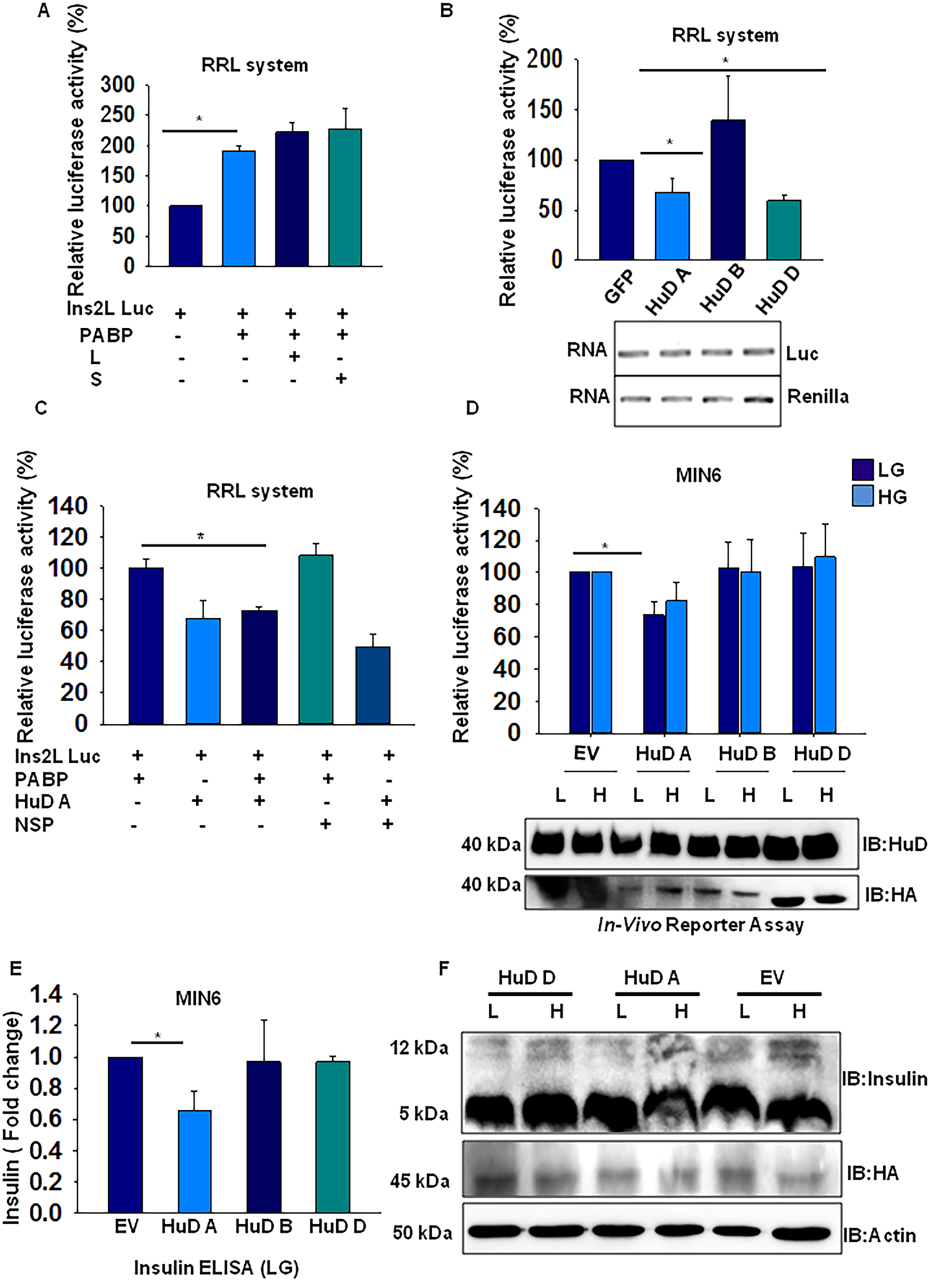
HuD-A inhibits insulin translation. *In vitro* translation assay of chimeric Ins2L-5’UTR-Luc-Ins2-3‘UTR **(A)** in presence of Long/Short (L/S) unlabeled RNA along with His-PABP **(B)** in presence of His-GFP or His-HuD A/B and D. Lower panel indicates the amount of luciferase and renilla RNA at the end of translation reaction detected by RT-PCR. Renilla was used as internal control for translation efficiency and His-GFP was used as nonspecific protein control and **(C)** in presence of recombinant PABP and/or HuD along with nonspecific protein (NSP). In all three experiments, the Luc/Renilla ratio was normalized and relative luciferase activity is expressed as a percentage of control and represented as bar graph. **(D)** MIN6 cells were transfected with HA-tagged HuD plasmid along with Long-5’UTR-Luc-Ins 3’UTR and Renilla luciferase plasmid. After 16 hours post transfection, the cells were treated with low glucose (2 mM) or high glucose (20 mM) for one hour and luciferase activity was measured. HuD expression was assessed by western blot analysis of the lysate (lower panel) **(E)** Secreted insulin levels in the low glucose condition media was assayed by ELISA in MIN6 cells overexpressing different HuD isoforms. The insulin readings were normalized to the control cells transfected with only empty vector (EV). **(F)** Intracellular proinsulin levels in low glucose condition were assayed in MIN6 cells by western blot analysis after overexpressing HA-tagged HuD isoforms along with empty vector control. HuD expression was assessed by Western blot analysis (lower panel). The graph represented in A, B, C and D are average of three experiments and the error bar represents the ± SEM and * indicates p<0.05 when compared with control.

To assess the function of PABP and HuD in insulin translation, we performed an *in vitro* translation assay. We used RNA containing luciferase ORF with long 5’UTR as reporter and performed translation reactions in the presence of recombinant proteins and renilla luciferase as normalizing RNA control. Although, PABP was able to stimulate the general translation of insulin 5’UTR as compared to non specific protein GFP (S5A Fig) but did not have any specific effect on insulin 5’UTR translation. The translation of both splice variants in presence of either long or short RNA remains unaffected as observed in decoy experiment (Fig 5A). Further, with respect to HuD we find that HuD isoforms A and D which binds to insulin 5’UTR are able to repress translation by about 30% and 40% respectively while B isoform had no significant effect on translation. This repression is solely due to translation as the transcript levels remained unaltered (Fig 5B). Further, we wanted to analyze what is the effect on translation of insulin when these molecules are together and we find that in RRL system PABP and HuD together inhibit the translation of insulin indicating that they form an inhibitory complex together on insulin 5’UTR (Fig 5C).

In order to examine the isoform specific regulation by HuD, *in vivo* luciferase reporter assay was performed by over expressing all three HuD isoforms in MIN6 cells along with the luciferase reporter constructs containing the insulin 5’UTRs. We found that only HuD-A repressed the translation of long 5’UTR reporter RNA significantly under low glucose condition while expression of HuD-B and HuD-D did not have any effect with respect to glucose stimulation (Fig 5D). Although this is in contrast to our *in vitro* data where HuD-D isoform was able to bind to insulin RNA and inhibits the translation of long variant (Fig 5B), suggesting that linker region (site for PTM, having multiple sites for phosphorylation) which is absent in HuD-D and present in HuD-A isoform may be important for translation repression under *in vivo* condition. In addition, we expressed HuD-B isoform in HEK cells as this cell line does not express any HuD isoforms [30] and we find that even in this system HuD-B seems to have no effect in insulin translation of both the splice variants and remain translationally inactive (S5B and S5C Fig).

Further, we measured the secreted insulin in these cells and found that HuD-A overexpression reduces the secreted insulin as compared to control, while HuD-B or HuD-D expression had no significant effect (Fig 5E). HuD-A overexpressing cells also showed a marginally reduced level of cell associated insulin in a glucose independent manner, as compared to HuD-D or empty vector control (upper band, Fig 5F). These finding clearly suggests that HuD-A isoform plays an important role in insulin translation repression.

### HuD and PABP interacts in RNA dependent manner and interaction is reduced in presence of glucose

**Fig 6:**
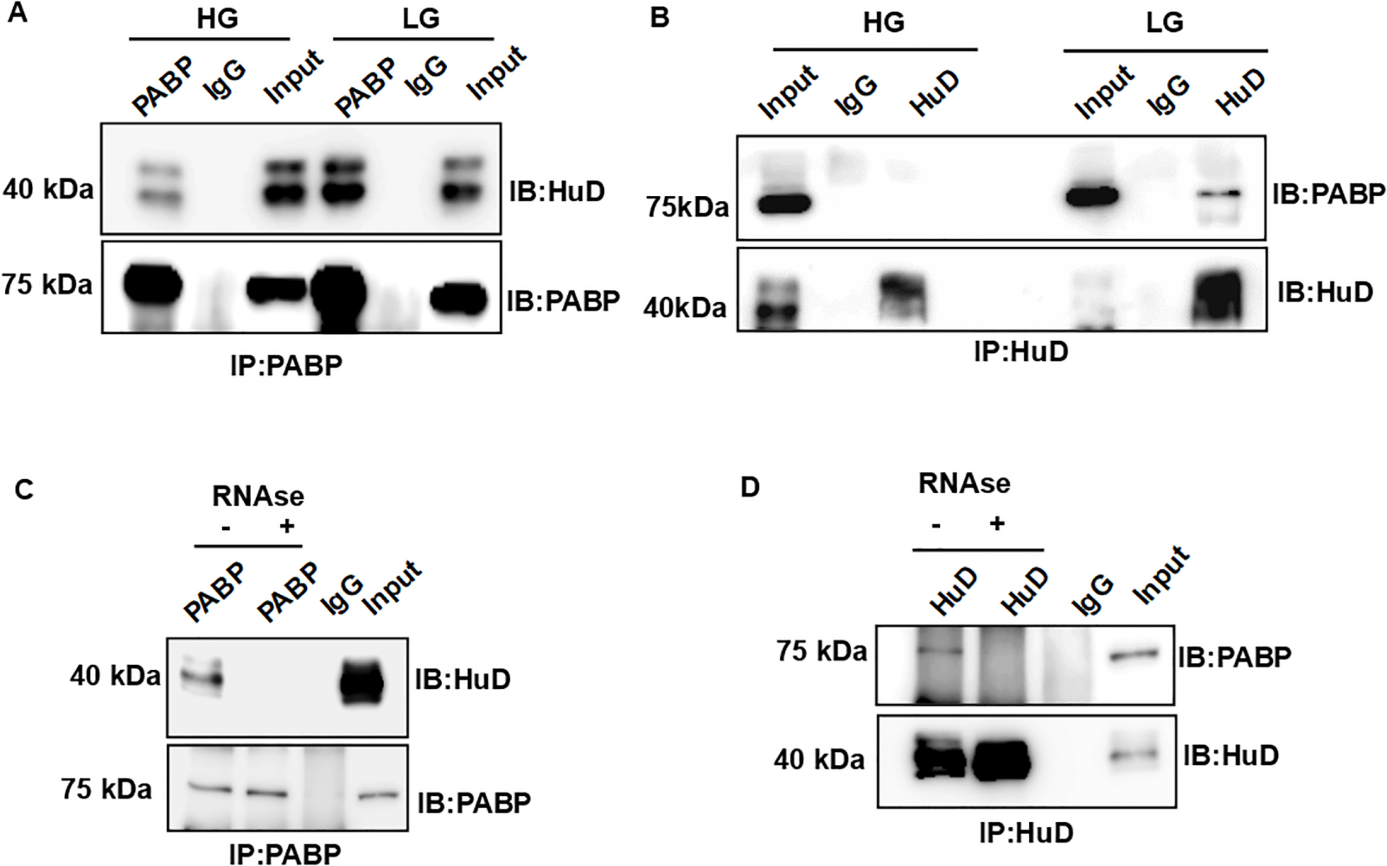
HuD and PABP interact in RNA-dependent manner under basal condition. **(A)** Immunoprecipitation of endogenous PABP from MIN6 cell lysates treated with low (LG) or high glucose (HG) condition and immunoblotted for HuD. **(B)** Reciprocal immunoprecipitation of endogenous HuD from low or high glucose treated cells and immunoblotted for PABP. **(C)** MIN6 cells treated with low glucose condition were lysed and the lysate was used to immunoprecipitate PABP or HuD in the presence (+) or absence of (-) RNase and probed for HuD or **(D)** PABP respectively. Rabbit IgG was used as a non specific antibody control for immunoprecipitation.

As we have shown earlier, HuD and PABP both bind to insulin 5’UTR at L2 region hence we wanted to determine whether they interact with each other. As discussed earlier, upon glucose stimulation, insulin translation is up regulated by 20 fold [31] and HuD-Ins2 mRNA complex is reduced and HuD is localized to P-bodies [17]. In order to analyze the interaction between PABP and HuD, we performed co-immunoprecipitation from insulinoma cells treated with low or high glucose. Co-immunoprecipitation of the extracts from low glucose treated cells with anti-PABP or anti-HuD antibodies showed PABP and HuD interact with each other and this interaction is clearly reduced under high glucose condition (Fig 6A and 6B). In addition, we have found that upon RNase treatment, the interaction between HuD and PABP lost in low glucose condition suggesting that this interaction is mediated by RNA (Fig 6C and 6D).

### The interaction between PABP and HuD is abrogated in presence of PDI

**Fig 7:**
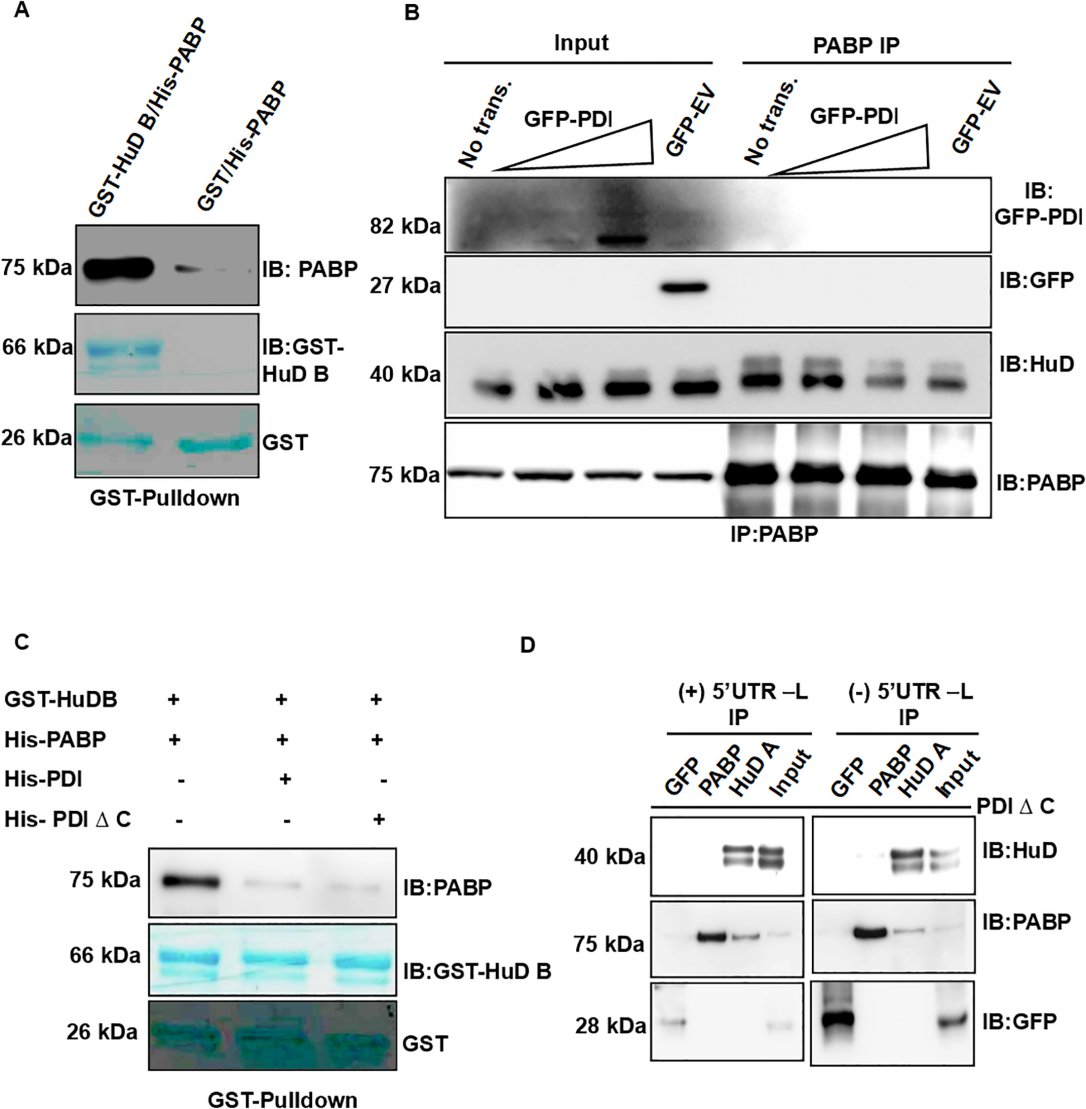
HuD and PABP interaction is regulated by PDI. **(A)** His-PABP was incubated with GST or GST-HuD B and GST-pull down was performed and analyzed by western blotting for presence of PABP, HuD-B or GST as indicated.**(B)** Immunoprecipitation of endogenous PABP was performed from the MIN6 cell transfected with GFP or GFP-PDI. The immunoprecipitate was analyzed for the presence of HuD, PABP, GFP or GFP-PDI by western blotting. **(C)** GST pull-down was performed from a protein mixture containing recombinant His-PABP, GST-HuD B, and His-PDI or His-PDIΔC (C-terminal deletion). The eluted proteins were analyzed for the presence of PABP, GST-HuD B and GST. **(D)** Recombinant purified HuD A, PABP, GFP and PDI Δ C were incubated with Ins2L-5’UTR (left panel) or without (right panel). The reaction was then immunoprecipitated with specific antibodies (indicated above the lanes) and the eluted fractions were analyzed by western blotting for the presence of HuD-A, PABP and GFP.

We carried out *in vitro* pull-down assays using recombinant PABP and HuD in the presence or absence of insulin 5’UTR in order to characterize this interaction further. To our surprise, we observed that recombinant PABP and HuD-B interacts with each other in the absence of RNA (Fig 7A). The precise reason for the differences observed between the native proteins and the recombinant protein is not clear. We hypothesized that presence cellular factors may weaken the interaction between PABP and HuD and insulin RNA may stabilize this interaction. Since PDI was previously shown to be the activator of insulin translation [9] hence we sought to determine if PDI could be the negative regulator of interaction between HuD and PABP. We assessed the HuD and PABP interaction in cells overexpressing GFP—PDI and found that presence of PDI reduces the interaction between HuD and PABP (Fig 7B). Further, GST-pull down experiments performed in presence of PDI or PDIΔC (mutant of PDI with insulin RNA binding activity) showed that interaction between recombinant PABP and HuD-B is reduced in presence of recombinant PDI or PDIΔC (Fig 7C) indicating that PDI interferes with this interaction. In addition, the role of insulin 5’UTR RNA in mediating this interaction was investigated by performing immunoprecipitation assay where we incubated recombinant HuD-A, PABP, and PDIΔC in the presence or absence of insulin 5’UTR and assessed the interaction of PABP and HuD by co-immnoprecipitation. Presence of insulin 5’UTR RNA increases the amount of PABP co-immunoprecipitated with HuD (Fig 7D, left panel), partially recapitulating the cellular interaction between PABP and HuD. We were unable to detect a significant amount of HuD in PABP immunoprecipitation experiments in the presence of PDIΔC (Fig 7D, right panel). Thus, our data indicate that PABP and HuD bind to the insulin 5’UTR and insulin mRNA remain translationally silent, however upon glucose stimulation, PDI inhibits the interaction of HuD with PABP resulting in activation of insulin translation. Together, these data suggest that the interaction between PABP and HuD and its association with insulin mRNA results in translation repression of insulin mRNA however upon glucose stimulation the interaction between PABP and HuD is abrogated by PDI which leads to the translation activation of insulin mRNA.

## Discussion

Glucose stimulation results in specific regulation of insulin splice variants in order to meet the excess insulin need [3]. It has been known for a long time that during the early phase of glucose stimulation (~1 h) insulin biosynthesis is primarily controlled at the translation level, but the exact mechanism has not been elucidated [32]. The interactions with a stem-loop structure of 5’UTR and the *trans-acting* factors seem to be important for translation regulation [33,34]. Thus, to understand the mechanistic details of insulin biosynthesis, it is important to identify the cytoplasmic factors that bind to insulin 5’UTR. We speculated that under low glucose/basal condition, stem-loop structure of 5’UTR is responsible for making a stable inhibitory complex with the *trans-acting* factors thus stalling the translational machinery. Here, we analyzed the interaction of specific factors that bind to the insulin 5’UTR under basal condition.

It has been reported that both the splice variants (L/S) of Ins2 are translated differentially [6]. In the present study, we show that specific factors in βTC6 and MIN6 cells bind differentially to the Ins2 splice variants. We identified PABP as one of the factors that associate differentially with mouse insulin2 mRNA splice variants. Further, fragment analysis of the 5’UTR shows that PABP binds to the middle L2 region. Secondary structure prediction suggests that it is part of loop-like structure in long isoform and double stranded stem-like structure in short isoform, hence we believe that differential binding of PABP is due to secondary structure of ‘A’ rich region. This observation is consistent with recent transcriptome-wide CLIP-Seq analysis where it was shown that PABP binds to 5’UTR region of many transcripts containing ‘A’ rich region in 5’UTR [16]. The role of PABP binding to the insulin 5’UTR is still not clear as *in vitro* translation assay shows that binding of PABP alone does not cause an alteration in translation efficiency. These results led us to believe that either post translation modifications or other factors may play a role in regulating differential translation of the insulin splice variants. Interestingly, binding experiments also suggest that HuD can bind to L2 and L4 regions of insulin 5’UTR. In neuronal cells, multiple HuD isoforms have been reported [19,21] however functional relevance and presence of some of the isoforms are still not clear. We studied three HuD isoforms A, B and D in insulinoma cells. The ‘N’ terminal sequence of HuD is important for RNA binding activity as B isoform which has identical sequence except for 7 amino acids at ‘N’ terminal is unable to bind while A and D isoforms having similar ‘N’ terminal is able to bind. It has been previously shown that the ‘N’ termini of HuD homolog (ELAV) are important for subcellular localization and RNA binding function in *Drosophila* [35]. We believe that this alternate ‘N’ terminal peptide has an important role in insulin translation regulation since in reporter assays we found that HuD-A was able to repress the translation and affect the insulin secretion and synthesis.

**Fig 8:**
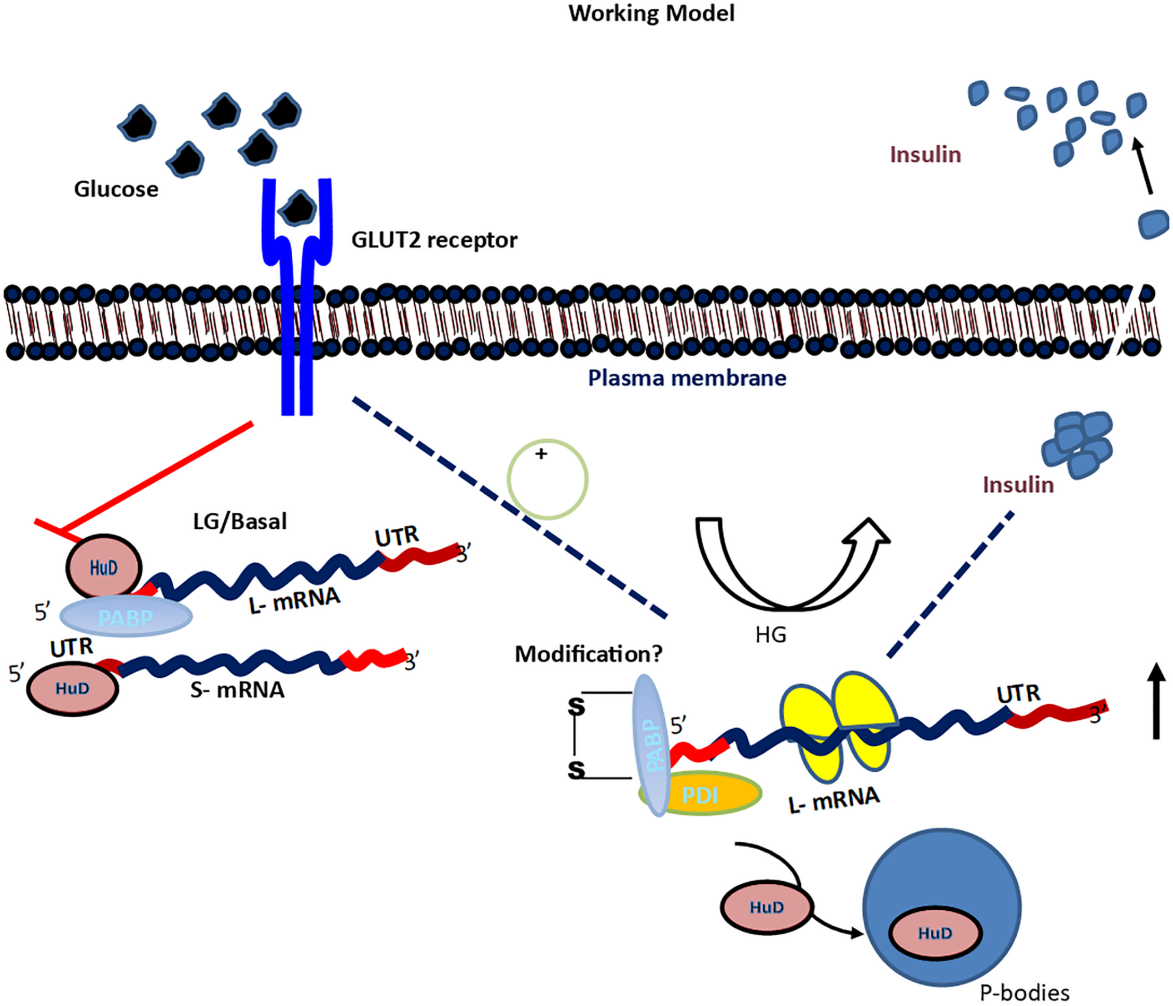
Mechanism of differential translation regulation of Ins2 splice variants. Under basal condition, PABP and HuD bind to long variant preferentially causing translation inhibition by making stable inhibitory complex. Upon glucose stimulation activated/phosphorylated PDI brings disulfide bond rearrangement in PABP thereby interfering with the interaction between HuD and PABP. This results in displacement of HuD from insulin transcript into P-bodies. The bound activator complex upregulates insulin translation and insulin is produced.

It has been reported that HuD also requires a poly-A-tail or cap structure to stimulate translation globally [36]. We propose that in case of insulin mRNA, PABP stabilizes the interaction of HuD containing inhibitory mRNP complex resulting in translation repression (Fig 8).

In summary, we showed that for the first time endogenous PABP and HuD interact with each other in RNA dependent manner and this interaction is diminished when the cells are stimulated with glucose. These data are consistent with the previous reports showing that HuD under high glucose condition is displaced from insulin 5’UTR and localizes to the P-bodies like structures [17]. We also observed that purified recombinant PABP and HuD-A proteins can interact without the requirement of RNA. This interaction is however inhibited in presence of PDI/PDIΔC (activators of insulin translation) suggesting that upon high glucose stimulation PDI could regulate HuD interaction with PABP and insulin mRNA. We propose that upon glucose stimulation PDI gets activated possibly by phosphorylation and is translocated to the cytoplasm where it rearranges the disulfide bonds of PABP [9] causing displacement of the inhibitory molecule such as HuD and enhancement of interaction with PABP and PDI (activating complex) shown in Fig 8. Overall, our study highlights the importance of HuD and PABP interaction through insulin 5’UTR resulting in differential regulation of insulin splice variants.

## Supporting information

S1 Fig Factors from βTC6 extract associate to short variant weakly. (TIF)

S2 Fig Cellular PABP associate with different affinity to Ins2 splice variants. (TIF)

S3 Fig PABP binds to L2 region of Ins2L 5’UTR. (TIF)

S4 Fig HuD B does not binds to Ins2L 5’UTR whereas HuD D binds differentially to both the splice variants at L2 and L4 region. (TIF)

S5 Fig PABP and HuD B do not cooperate in specific translation. (TIF)

S1 Table List of primers used in PCR and cloning. (DOCX)
