# Supplementary figures and images for: Interaction of HuDA and PABP at 5'UTR of mouse insulin2 regulates insulin biosynthesis
Source: PLoS One. 2018 Mar 28;13(3):e0194482. doi: 10.1371/journal.pone.0194482 (PMC5874046; doi:10.1371/journal.pone.0194482)

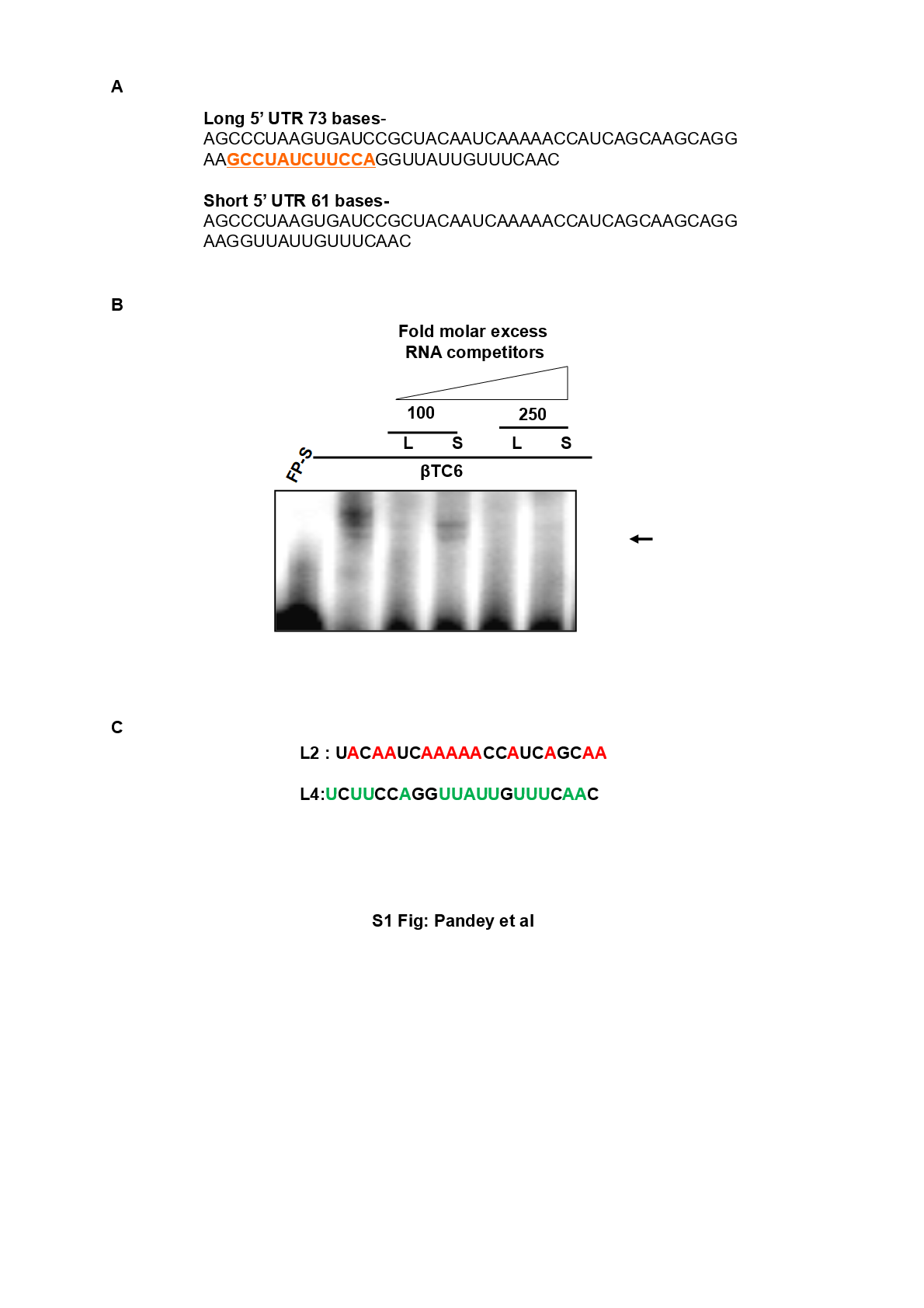

Supplement: S1 Fig — (TIF) [file pone.0194482.s001.tif]

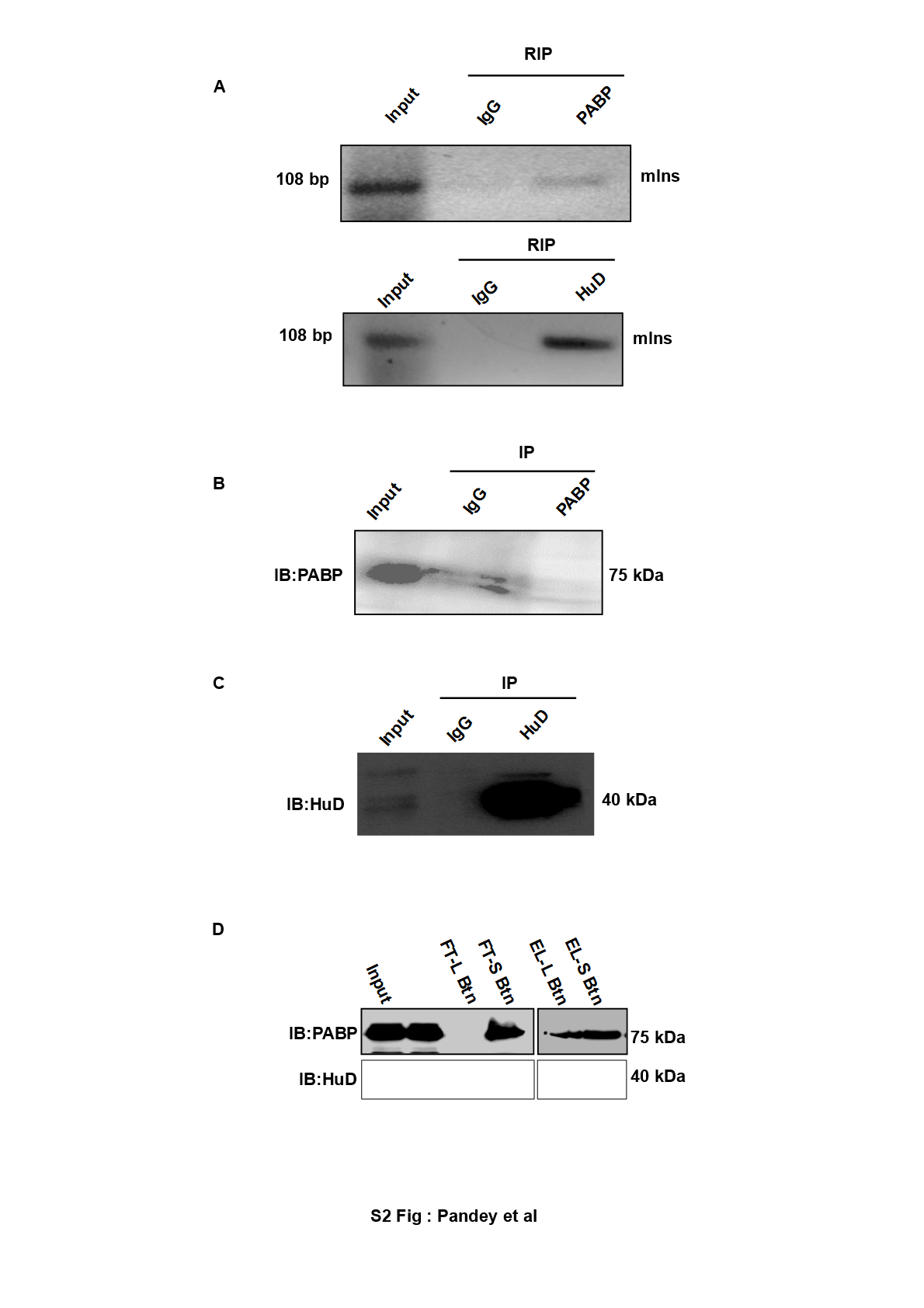

Supplement: S2 Fig — (TIF) [file pone.0194482.s002.tif]

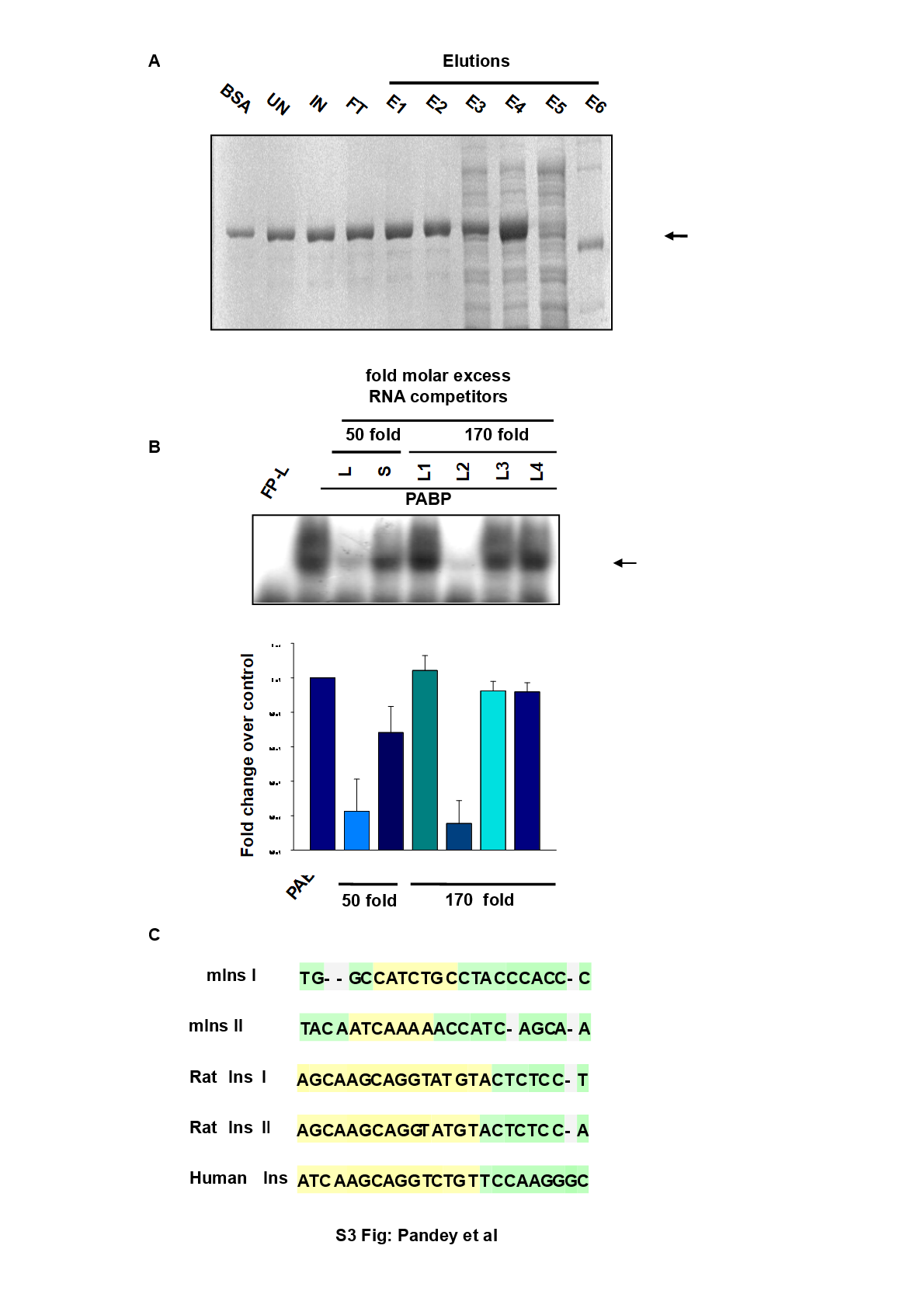

Supplement: S3 Fig — (TIF) [file pone.0194482.s003.tif]

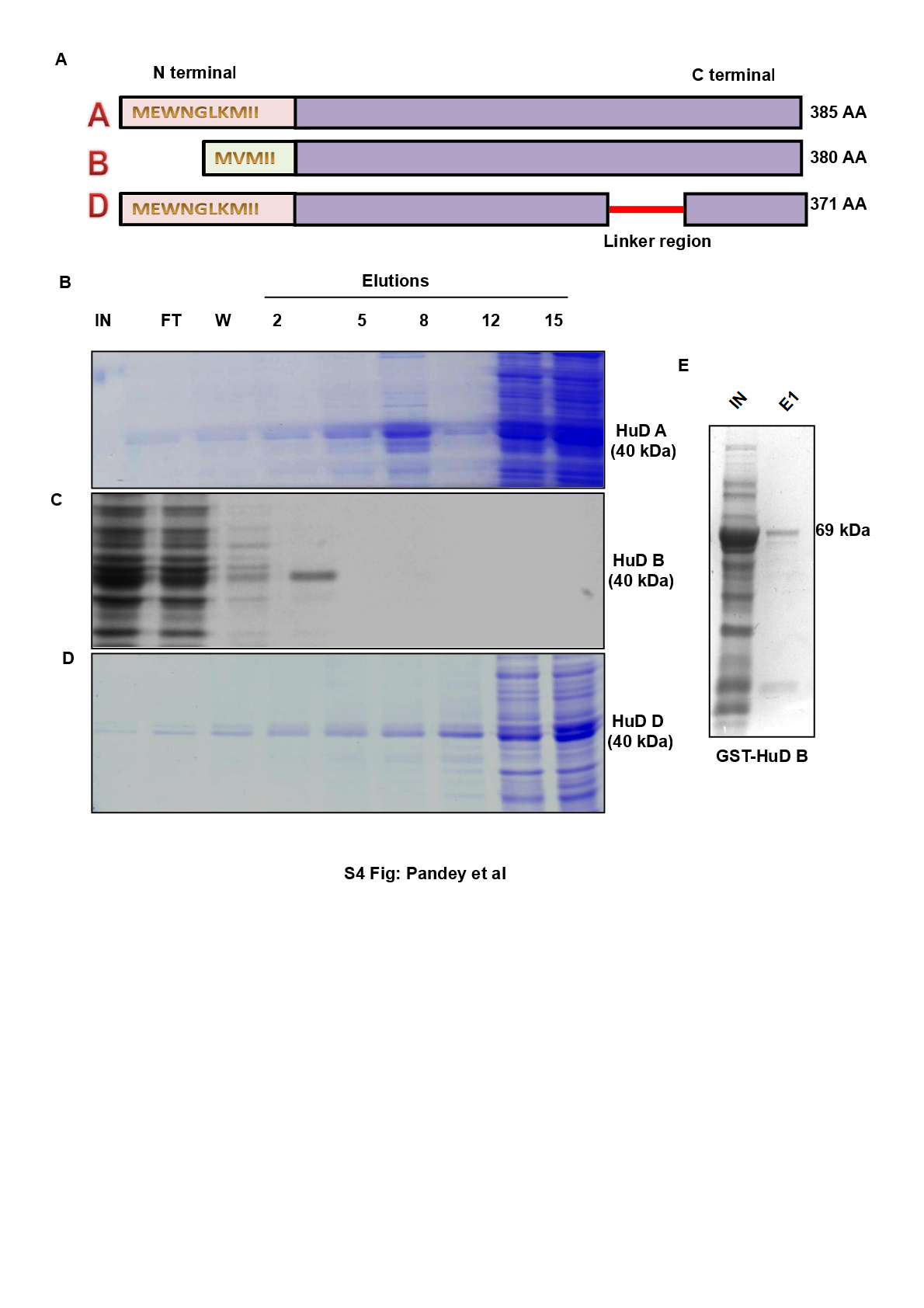

Supplement: S4 Fig — (TIF) [file pone.0194482.s004.tif]

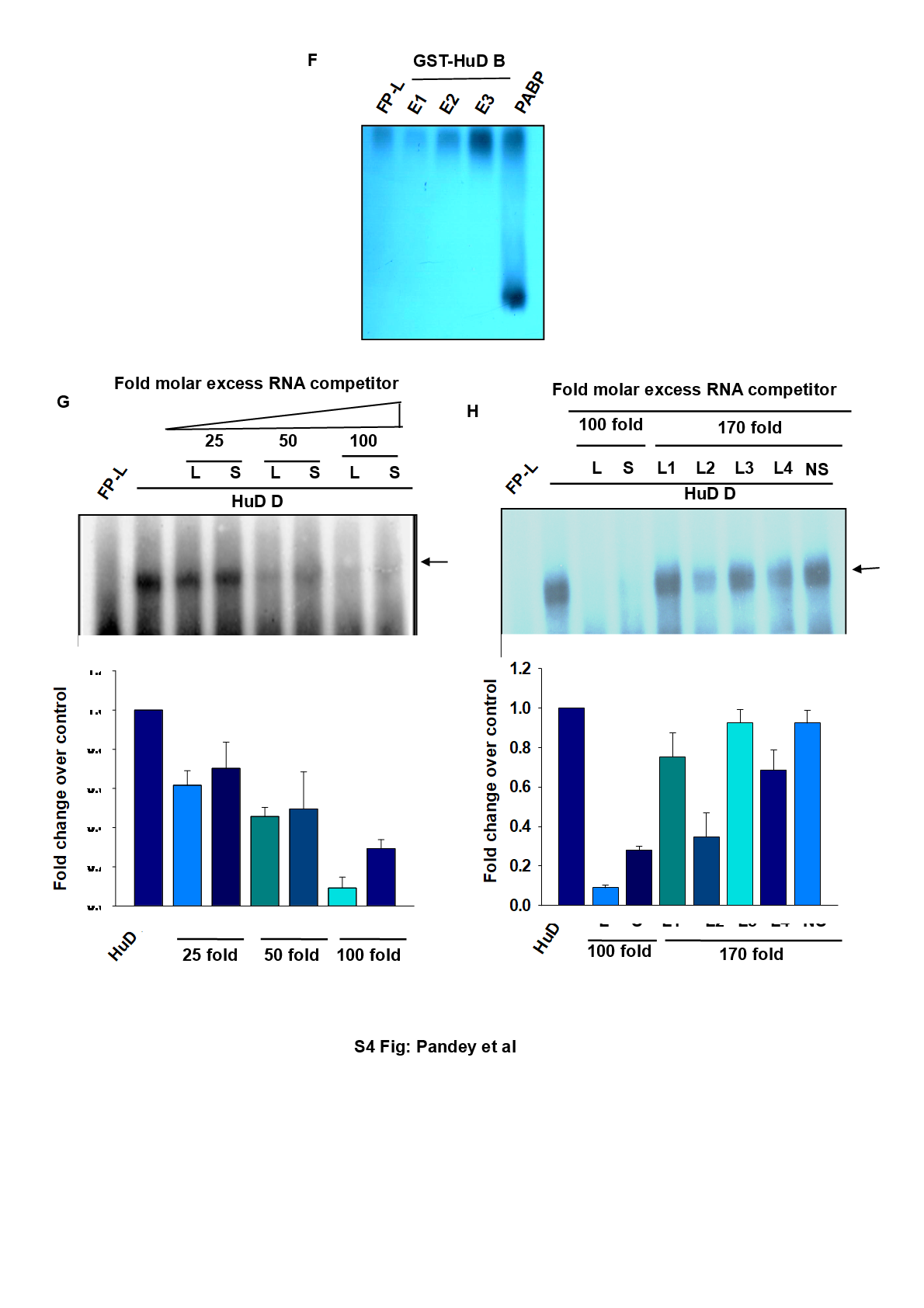

Supplement: S5 Fig — (TIF) [file pone.0194482.s005.tif]
