# Supplementary material for: Interaction of HuDA and PABP at 5'UTR of mouse insulin2 regulates insulin biosynthesis
Source: PLoS One. 2018 Mar 28;13(3):e0194482. doi: 10.1371/journal.pone.0194482 (PMC5874046; doi:10.1371/journal.pone.0194482)
Supplement: S1 Table — (DOCX) [file pone.0194482.s006.docx]

***Interaction of HuDA and PABP at 5’UTR of mouse insulin2 regulates insulin biosynthesis***

**Poonam R. Pandey^1,2^, Rucha D. Sarwade^1,2^, Abdul Khalique^1,2^, Vasudevan Seshadri^1^***

**Authors Affiliation:**

**^1^** National Centre for Cell Science, Ganeshkhind, Pune 411007, India.

**^2^** Department of Biotechnology, Savitribai Phule Pune University, Ganeshkhind, Pune 411007, India.

*** Correspondence to:** Email: [seshadriv@nccs.res.in](mailto:seshadriv@nccs.res.in)

**Funding Details:**

This work is supported by a grant to V. S. from Department of Biotechnology, Government of India (BT/PR14109/BRB/10/812/2010) and intramural funds from National Centre for Cell Science (NCCS), Pune, India. The funders had no role in study design, data collection, and analysis, decision to publish, or preparation of the manuscript.

**Disclosure of potential conflicts of interest**

No potential conflicts of interest were disclosed.

**Supporting figure legends:**

**S1 Fig. Factors from βTC6 extract associate to short variant weakly. (A)** Sequence of long (Ins2L) and short 5’UTR (Ins2S), colored sequences in Ins2L shows spliced 12 bases from Ins2 gene. **(B)** Competitive RNA-EMSA using radiolabeled short 5’UTR in presence of the molar excess of unlabeled Ins2L and Ins2S competitors incubated with βTC6 extract. **(C)** Sequence analysis of fragments of long 5’UTR of Insulin (L2 and L4), red highlighted sequences shows stretch of A residues in L2 and sequences in green shows AU rich region in L4.

**S2 Fig. Cellular PABP associate with different affinity to Ins2 splice variants.** UV crosslinked MIN6 cells were used for studying the interaction of PABP and HuD with insulin transcript by RIP followed by RT-PCR where IgG is used as negative control **(A)** RT-PCR analysis of insulin RNA associated with PABP (top panel) or HuD (lower panel) amplified using mouse insulin primers. Immunoprecipitation of (**B)** PABP and (**C)**HuD from MIN6 lysate subjected to RIP analysis. **(D)** Western blot of PABP/HuD bound with L-Biotin (L-Btn) and S-Biotin (S-Btn) RNA. After collecting flow through (FT) from L and S biotinylated RNA, eluted fractions (EL) from L and S were probed for PABP (Upper panel) and HuD (lower panel).

**S3 Fig. PABP binds to L2 region of Ins2L 5’UTR. *(*A)** 10 µg of soluble protein from uninduced and induced bacterial culture along with His-PABP after purification with Ni-NTA agarose beads were resolved on 10% SDS-PAGE followed by Coomassie blue staining. ***(*B)** Competitive RNA-EMSA with His-PABP in presence of equal fold molar excess of unlabeled RNA (L, S, L1, L2, L3, L4) using radiolabeled Ins2L along with densitometric quantification (mean + SEM, n=3; *, p < 0.004 as compared to when no competitors were present). ***(*C)** T-COFFEE sequence alignment of L2 region of Ins2L across species.

**S4 Fig. HuD-B does not bind to Ins2L 5’UTR whereas HuD-D binds differentially to both the splice variants at L2 and L4 region. (A)** Schematic illustration of HuD isoforms protein with different “N” terminal sequence indicated. Red lines show the absence of linker region in HuD-D (Boxes are not drawn to scale). Fractions of induced bacterial culture along with purified HuD protein (10 µg) after purification with Ni-NTA column were resolved on 10% SDS-PAGE followed by Coomassie blue staining. **(B)** His-HuD A purification **(C)** His-HuD B purification **(D)** His-HuD D purification. **(E)** GST-HuD B induced supernatant along with purified GST-HuD B after purification with glutathione sepharose beads*.* **(F)** RNA-EMSA with purified GST-HuD B incubated with radiolabeled Ins2L 5’UTR **(G)** Competitive RNA-EMSA with purified recombinant HuD-D with radiolabeled Ins2L 5’UTR in presence of a molar excess of unlabeled L/S competitors and **(H)** in presence molar excess of unlabeled long fragments *viz* (L1, L2, L3, L4) RNA along with densitometry quantification. (Mean + SEM, n=3; #, p <0.05 values indicated are compared to short RNA (100 fold), *, p <0.036 and **, p <0.006 values are compared when no competitors were present).

**S5 Fig. PABP and HuD-B do not cooperate in specific translation. (A)** *In vitro* translation assay of chimeric Ins2L 5’UTR Luc-Ins2-3‘UTR in presence of His-PABP and an equal amount of His-GFP. Renilla luciferase RNA was used as internal control for translation efficiency and His-GFP was used as nonspecific protein control. **(B**) *In vivo* translation assay in HEK 293 cells by overexpressing HuD B cotransfected with Ins2L 5’UTR-Luc-Ins 3’UTR and **(C)** Ins2S 5’UTR-Luc-Ins 3’UTR along with Renilla construct. Western blot indicates the overexpression efficiency of HuD B isoform. The Luc/Renilla ratio was normalized with respect to control and relative luciferase activity is expressed as in percentage and represented into bar graph (mean + SEM, n=3).

**Supporting table S1**

| 1. **11.** | **Ins2L-5’UTR sense** | **AGCTTAGCCCTAAGTGATCCGCTACAATCAAAAACCATCAGCAAGCAGGAAGCCTATCTTCCAGGTTATTGTTTCAACA** |
| --- | --- | --- |
| **2.** | **Ins2S-5’UTR sense** | **AGCTTAGCCCTAAGTGATCCGCTACAATCAAAAACCATCAGCAAGCAGGAAGGTTATTGTTTCAACA** |
| **3.** | **Ins2L-AS-T7**  **sequence** | **GTTGAAACAATAACCTGGAAGATAGGCTTCCTGCTTGCTGATGGTTTTTGATTGTAGCGGATCACTTAGGGCTCCCTATAGTGAGTCGTATTAGCAT** |
| **4.** | **Ins2S-AS-T7**  **sequence** | **GTTGAAACAATAACCTTCCTGCTTGCTGATGGTTTTTGATTGTAGCGGATCACTTAGGGCTCCCTATAGTGAGTCGTATTAGCAT** |
| **5.** | **Ins-RT-F** | **CCCCTGCTGGCCCTGCT** |
| **6.** | **Ins-RT-R** | **CTCCCCACACACCAGGTAG** |
| **7.** | **Ins2L-F** | **CATCAGCAAGCAGGAAGCCTATC** |
| **8.** | **Ins2L-R** | **CCCACACACCAGGTAGAGAG** |
| **9.** | **Ins2-S-F** | **CAGCAAGCAGGAAGGTTATTGT** |
| **10.** | **Ins2-S-R** | **ACCAGGTGGGAACCACAAAG** |
| **11.** | **L1T7AS** | **TTGTAGCGGATCACTTAGGGCTCCCTATAGTGAGTCGTATTAGCAT** |
| **12.** | **L2T7AS** | **TTGCTGATGGTTTTTGATTGTACCCTATAGTGAGTCGTATTAGCAT** |
| **13.** | **L3T7AS** | **GAAGATAGGCTTCCTGCTTGCTCCCTATAGTGAGTCGTATTAGCAT** |
| **14.** | **L4T7AS** | **GTTGAAACAATAACCTGGAAGACCCTATAGTGAGTCGTATTAGCAT** |
| **15.** | **HuD-B-F** | **ACTGGGATCCGATGGTTATGATAATTAGCACC** |
| **16.** | **HuD-R** | **ACCGCTCGAGGGATTTGTGGGCTTTGTTGGT** |
| **17.** | **HuD-ApGex** | **CTTCGGATCCATGGAGTGGAATGGCTTG** |
| **18.** | **HuD-BpGex** | **CTTCGGATCCATGGTTATGATAATTAGCACC** |
| **19.** | **HuD-A-F** | **CTTC*G*GATCCACAGATGGAGTGGAATGGCTTG** |
